# Functional characterization of *SDHB* variants clarifies hereditary pheochromocytoma and paraganglioma risk and genotype-phenotype relationships

**DOI:** 10.1172/JCI198165

**Published:** 2025-11-18

**Authors:** Sooyeon Lee, Leor Needleman, Julie Park, Rebecca C. Schugar, Qianjin Guo, James M. Ford, Justin P. Annes

**Affiliations:** 1 Department of Medicine, Division of Endocrinology and; 2 Department of Medicine, Division of Oncology, Stanford University, Stanford, California, USA.; 3 Stanford ChEM-H and Endocrine Oncology, Stanford University Cancer Institute, Stanford, California, USA.

**Keywords:** Endocrinology, Genetics, Genetic diseases, Molecular diagnosis, Neuroendocrine regulation

## Abstract

Hereditary pheochromocytoma and paraganglioma (hPPGL) is caused by pathogenic mutations in succinate dehydrogenase (SDH) genes, commonly *SDHB*. However, over 80% of *SDHB* missense variants are classified as variants of uncertain significance (VUS), limiting clinical interpretation and diagnostic utility of germline testing. To provide functional evidence of SDHB allele pathogenicity or benignity, we developed a cellular complementation assay that quantifies intracellular succinate/fumarate ratios as a readout of SDH enzymatic activity. This assay reliably distinguished pathogenic from benign alleles with high fidelity, outperforming and complementing computational predictions. Functional assessment of patient-derived VUS alleles supported reclassification of 87% of tested variants and revealed that mutations in the iron-sulfur cluster domain were amorphic, while those at or beyond the C-terminal residue Tyr273 retained function. Variants associated with Leigh syndrome retained activity, consistent with their biallelic inheritance and distinct pathogenic mechanisms from *SDHB*-related tumorigenesis. Notably, hypomorphic pathogenic *SDHB* variants correlated with increased head and neck paraganglioma occurrence, revealing a genotype-phenotype relationship. Functional characterization of *SDHB* missense variants supports clinical classification, informs hPPGL risk stratification, and has immediate diagnostic impact.

## Abstract 2

Functional testing of SDHB missense variants improves clinical classification, supports hereditary pheochromocytoma and paraganglioma risk stratification, and informs early diagnosis.

## Introduction

Pheochromocytoma (PCC) and paraganglioma (PGL) are tumors that arise from chromaffin cells of the adrenal medulla or extra-adrenal paraganglia, respectively (1). Paragangliomas that arise from parasympathetic head and neck paraganglia cells (hnPGL) are separately categorized. Collectively referred to as PPGL, these tumors have a prevalence of approximately 1 in 3,600 individuals (2, 3) and typically present in the third-to-fifth decades of life (4, 5). While PPGLs are often amenable to surgical resection, 10%–30% of cases demonstrate metastatic progression, most commonly to lymph nodes, bone, and/or lungs (6). The limited efficacy of metastatic PPGL treatments highlights the need for early diagnosis to improve clinical outcomes (7, 8). Moreover, PPGLs exhibit one of the highest rates of heritability among all neoplastic disease, with 25%–40% of cases attributable to germline mutations (9, 10). In fact, the strong genetic basis of PPGL justifies universal germline testing of all affected individuals so at-risk patients and their family members can benefit from counseling and lifelong clinical surveillance, even when asymptomatic (11–14).

Among the many PPGL susceptibility genes, the succinate dehydrogenase (SDH) gene family accounts for a substantial proportion of cases (1, 15). Of these, the SDH subunit gene *SDHB* is one of the most prevalent and is associated with increased risk for recurrence and metastasis (16–18). The estimated pathogenic/likely pathogenic *SDHB* allele frequency is approximately 1 in 8,000 and surveillance in carriers of *SDHB* pathogenic variants facilitates early tumor detection and reduces mortality (11, 12). Notably, interpretation of *SDHB* missense variants remains a major clinical challenge.

Currently, IHC for SDHB serves as a standard pathologic tool for aiding clinical interpretation of germline variants of *SDHx* genes (*SDHA, SDHB, SDHC, SDHD*, or *SDHAF2*) (19, 20); however, presymptomatic individuals harboring VUS alleles do not benefit from this methodology. Additionally, widespread adoption of germline “cancer panel” testing has increased incidental identification of *SDHB* variants, many of which are nonactionable VUS alleles — leaving families at risk for potentially preventable disease consequences. Interpretation is further complicated by incomplete penetrance (approximately 25%–35% lifetime PPGL risk for *SDHB*) and a high proportion of private mutations, for which little segregation or population-level evidence is frequently available (2, 12). Hence, there is a need for biologically relevant tools to clarify *SDHB* variant pathogenicity and enable appropriate clinical management.

SDH or complex II (CII), is a mitochondrial enzyme that plays a dual role in the tricarboxylic acid (TCA) cycle and electron transport chain (ETC). The SDH complex consists of 4 nuclear-encoded subunits (SDHA–D) that embed in the inner mitochondrial membrane (21). SDH catalyzes the oxidation of succinate to fumarate, generating FADH_2_, which donates electrons to ubiquinone in the ETC (22). *SDHx* genes typically behave like classical tumor suppressor genes, wherein tumorigenesis occurs through inheritance of a pathogenic allele and stochastic loss of heterozygosity or silencing of the WT allele, resulting in loss of SDH function in the tumor (23). Consequently, *SDHx*-associated tumors exhibit dramatic succinate accumulation — often 100-fold above normal levels (24, 25) — and elevated succinate-to-fumarate ratios (26, 27). Although a complete mechanistic understanding of *SDHx* tumorigenesis remains elusive, succinate is considered to be an “oncometabolite,” a family of TCA cycle metabolites that accumulate as a consequence of enzyme disruption and contribute to oncogenic signaling (28, 29). Within the oncometabolite paradigm, succinate promotes tumorigenesis via competitive inhibition of 2-oxoglutarate–dependent dioxygenases, including HIF prolyl-hydroxylases and TET demethylases, leading to metabolic, transcriptional, and epigenetic reprogramming (30–33). Given that *SDHx*-driven tumorigenesis depends on impaired succinate oxidation, the succinate-to-fumarate ratio serves as a mechanistically informed biomarker for interpreting *SDHx* allele pathogenicity.

To address the unmet need to clarify *SDHB* VUS interpretation, we developed a robust, scalable assay that quantifies SDH enzymatic activity via succinate/fumarate ratios. This approach aligns with American College of Medical Genetics and Genomics (ACMG), the Association for Molecular Pathology (AMP), and Clinical Genome Resource (ClinGen) variant interpretation guidelines, which recognize validated functional assays as strong evidence for pathogenicity (PS3) or benignity (BS3) (34–36). In this study, we report an SDHB-specific assay that distinguishes pathogenic from benign PPGL-risk variants.

## Results

### Generation and validation of a functional complementation assay for SDHB variants.

**Figure 1:**
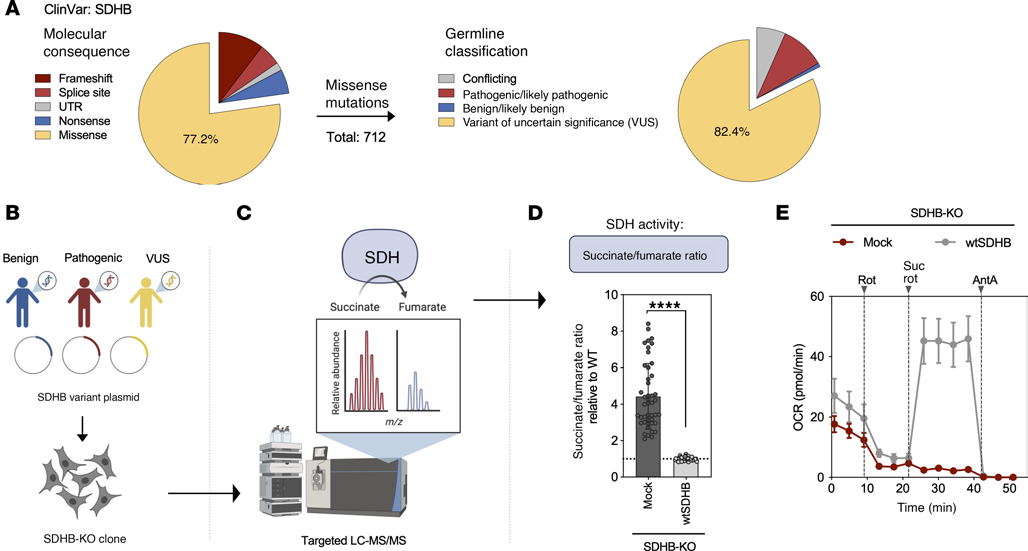
A functional assay to characterize *SDHB* missense variants. (**A**) Percentages of molecular events (left) and germline classifications (right) for *SDHB* variants annotated in ClinVar (accessed July 7, 2025). (**B**–**D**) Overview of the SDHB complementation assay. (**B**) *SDHB* variants (benign, pathogenic, VUS) were transfected into SDHB-knockout HEK293 cells (SDHB-KO). (**C**) Succinate and fumarate levels were quantified by targeted LC-MS/MS. (**D**) Succinate/fumarate ratios measured in mock-transfected and WT SDHB (wtSDHB-transfected) SDHB-KO cells, *n* = 45 biological replicates from more than 10 independent experiments. (**E**) Oxygen consumption rate (OCR) assessing Complex II activity in mock- and wtSDHB-transfected SDHB-KO cells. Suc, Succinate. Rot, Rotenone. AntA, Antimycin A. Mean ± SD are shown. Unpaired 2-tailed Student’s *t* test used in **D**, ****P < 0.0001.

ClinVar reported 712 germline *SDHB* missense variants, of which 82% were classified as VUS (Figure 1A). This high proportion of VUS presents a major clinical challenge, as they are nonactionable for patient management and surveillance. To address this gap, we developed a biochemical complementation assay to directly evaluate the functionality of *SDHB* missense variants.

Using CRISPR/Cas9-based genome editing, we generated *SDHB*-knockout HEK293 cells (SDHB-KO), which were confirmed by Western blotting and immunostaining (Supplemental Figures 1 and 2; supplemental material available online with this article; https://doi.org/10.1172/JCI198165DS1). This approach enabled expression of individual *SDHB* variants in a null background (Figure 1B) and direct quantification of succinate and fumarate levels via targeted LC-MS/MS (Figure 1C). To confirm the effectiveness of our *SDHB* complementation assay, we transfected WT human *SDHB* (wtSDHB) into SDHB-KO cells. Reintroduction of wtSDHB reduced intracellular succinate levels (Supplemental Figure 2, B and C), significantly lowering the succinate-to-fumarate ratio (Figure 1D). Herein, the succinate-to-fumarate ratio normalized to wtSDHB (referred to as Succinate/Fumarate Ratio) is used as a primary metric for assessing *SDHB* variant function. To further support the use of this readout as a proxy for SDH enzymatic activity, we measured complex II–specific oxygen consumption rates (OCR) using the Seahorse assay. SDHB-KO cells exhibited a marked loss of complex II activity, which was restored upon expression of wtSDHB (Figure 1E). These findings confirmed that the succinate/fumarate ratio corresponded to mitochondrial SDH function and its use for evaluating *SDHB* variant function.

**Figure 2:**
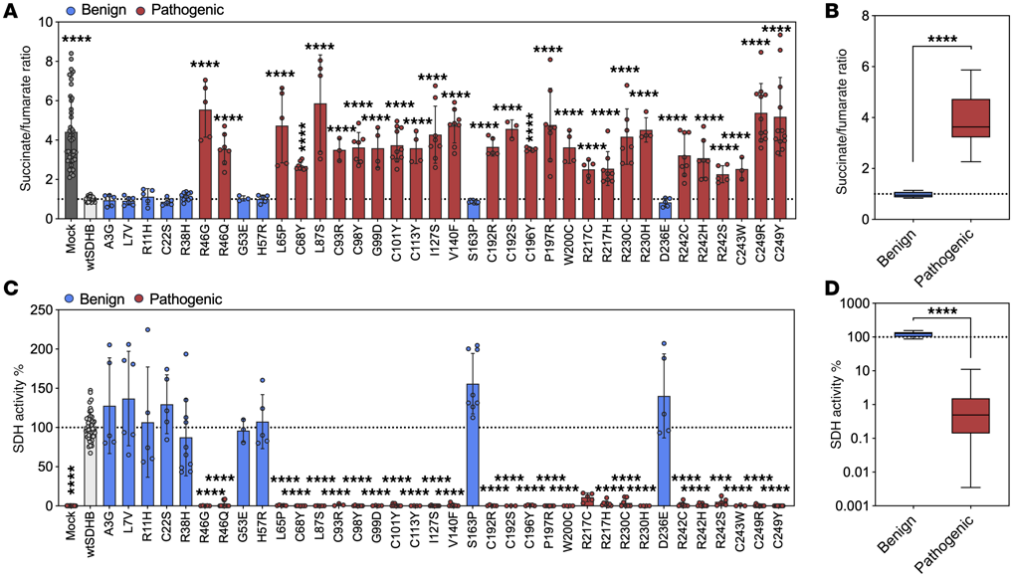
Functional discrimination of classified *SDHB* variants via succinate/fumarate ratio and SDH enzymatic activity. (**A**) Succinate/fumarate ratios for 36 *SDHB* missense variants (9 benign, blue; 27 pathogenic, red) transfected into SDHB-KO cells. (**B**) Summary of the ratios grouped by classification. (**C**) SDH activity percentage (derived from succinate/fumarate ratios) calibrated to wtSDHB (100%) and mock (0.1%) for each variant. Statistical comparisons are shown for pathogenic variants relative to wtSDHB to highlight loss of SDH function. (**D**) Summary of activity values grouped by classification. Each point represents a biological replicate. Each variant was tested in at least 2 independent experiments. Mean ± SD are shown. Ordinary 1-way ANOVA used in **A** and **C** (Dunnett’s multiple tests compared to wtSDHB with lognormal distribution assumption) and unpaired 2-tailed Student’s *t* test used in **B** and **D**, ****P < 0.0001; ***P < 0.001; **P < 0.01; *P < 0.05.

Next, we evaluated the performance of our assay using a reference set of variants with established clinical classifications. We characterized 36 *SDHB* missense variants reported in ClinVar as benign/likely benign (“benign”) or pathogenic/likely pathogenic (“pathogenic”). All the benign variants yielded succinate/fumarate ratios comparable to the wtSDHB construct (Figure 2A), indicating functional rescue. In contrast, all 27 pathogenic variants showed elevated succinate/fumarate ratios, consistent with amorphic or severely hypomorphic allele activity (Figure 2A). Benign variants had a mean succinate/fumarate ratio of 0.97, while pathogenic variants had a significantly higher mean of 3.894 (Figure 2B). The expression of experimentally tested variants was confirmed by Western blot (Supplemental Figure 3).

To improve interpretability and standardization across experiments, we converted succinate/fumarate ratios into relative SDH activity using an exponential model calibrated to mock- and wtSDHB-transfected SDHB-KO cells. Each experiment was scaled such that the mean of wtSDHB-transfected replicates was set to 100% activity and mock-transfected replicates to 0.1% activity. Similar to the succinate/fumarate ratio–based results, benign variants restored SDH activity to WT-like levels, whereas pathogenic variants exhibited profound loss of function, with a mean activity of 1.30% (IQR: 0.14%–1.54%) and the highest observed value of 11.06% (Figure 2, C and D). These results demonstrated that our assay faithfully differentiated pathogenic from benign *SDHB* variants based on their impact on SDH enzymatic activity.

### Logistic modeling for pathogenicity prediction of SDHB variants.

**Figure 3:**
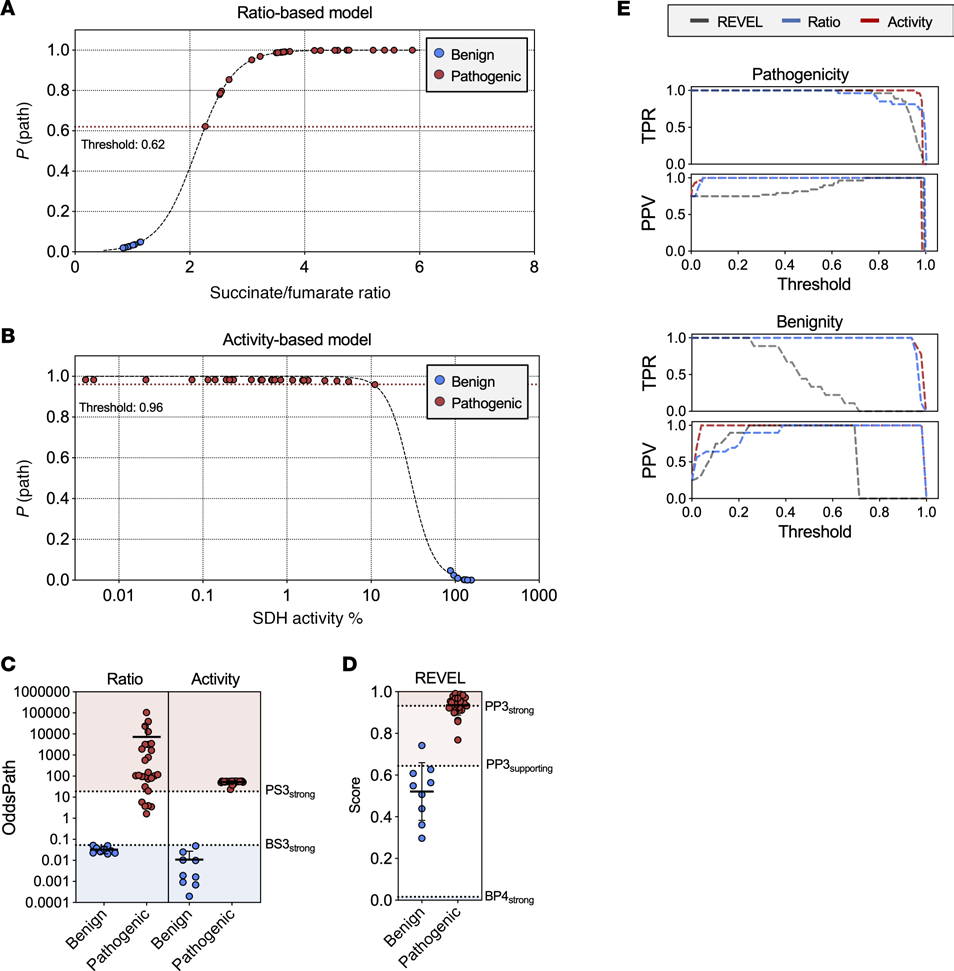
Functional model outperforms REVEL in discriminating pathogenic from benign SDHB variants. (**A**) Predicted probability of pathogenicity [*P(path)*] plots for classified *SDHB* variants generated using logistic regression models based on succinate/fumarate ratio or (**B**) normalized SDH activity. Thresholds are represented by red dotted line. (**C**) OddsPath scores derived from ratio- and activity-based models. Cutoffs for strength of functional evidence are shown: PS3_strong_ (> 18.7), dashed line/pink shading; BS3_strong_ (< 0.053), dotted line/blue shading. (**D**) REVEL scores and associated ACMG/AMP evidence assignments: PP3_strong_ (> 0.932), dashed line/pink shading; BP3_strong_ (< 0.016), dotted line/blue shading. (**E**) True positive rate (TPR) and positive predictive value (PPV) for models across thresholds. Mean ± SD are shown.

To leverage observed functional distinctions for variant classification, we trained separate logistic regression models using either succinate/fumarate ratio or SDH activity determined using the benign and pathogenic variants. Each model was implemented as a scikit-learn pipeline with feature standardization, and regularization strength (C) was optimized using GridSearchCV to minimize average log loss across 5-fold cross validation. This approach prioritized probability calibration over classification accuracy, consistent with previous modeling (37). Pathogenic variants were assigned as the positive class, and the trained models were used to generate *P*(*path*) values for each variant based on either the succinate/fumarate ratio (Figure 3A) or SDH activity (Figure 3B). In the ratio-based model, pathogenic variants had a mean *P(path)* of 0.950. SDHB^R242S^ exhibited the lowest *P(path*) value of 0.622, which is reflective of the moderate succinate/fumarate ratio relative to other pathogenic variants. In comparison, the activity-based model yielded uniformly high *P(path)* values for all pathogenic variants, resulting in a more stringent threshold of 0.960. Both models separated pathogenic from benign variants, with the activity-based model achieving a more distinct separation between groups. These findings suggested that the calculated SDH activity might serve as a more discriminating predictor of pathogenicity.

To evaluate the utility of each model within the ACMG/AMP variant interpretation framework, we converted *P(path)* values into odds of pathogenicity (OddsPath), enabling assignment of functional evidence strength to each variant. This approach allows compatibility with BS3 (evidence against pathogenicity) and PS3 (evidence for pathogenicity) criteria (34). All benign variants met the threshold for BS3_strong_ evidence in both the ratio- and activity-based models (Figure 3C). Among pathogenic variants, the ratio-based model yielded a broader range of OddsPath values and evidence strength, ranging from PS3_strong_ (SDHB^L87S^) to unclassified (SDHB^R242S^). In contrast, the activity-based model classified all pathogenic variants as PS3_strong_. The reduced variability of the activity-based model reflects additional normalization to the simultaneously performed mock-transfection condition.

According to ClinGen recommendations, computational models can provide functional evidence supporting either pathogenicity (PP3) or benign impact (BP4), with strength of evidence ranging from supporting to strong, depending on the score thresholds and validation of the tool (38). The REVEL ensemble score is the in silico predictor currently endorsed by ClinGen Variant Curation Expert Panels (VCEPs) (39). Using established thresholds (38), REVEL classified all pathogenic variants as meeting PP3-level evidence, with 16 receiving PP3_strong_ and the remaining 11 assigned PP3_moderate_ or PP3_supporting_ (Figure 3D). In contrast, REVEL failed to correctly identify benign variants as benign; none met BP4 evidence. Instead, 8 variants were unclassified, and 1 (SDHB^D236E^) was misclassified as PP3_supporting_, indicating a false positive. As a result, REVEL demonstrated good sensitivity and specificity, yielding high true positive rates (TPR) and positive predictive values (PPV) for predicting variant pathogenicity; however, REVEL performed relatively poorly in predicting variant benignity (Figure 3E). By contrast, our functional models performed extremely well at predicting both pathogenicity and benignity, clearly outperforming REVEL in the latter. Notably, unlike REVEL, which failed to assign BP4 to any benign variant, our models accurately identified and classified benign variants with high specificity (Figure 3E). These findings highlight the enhanced predictive power and clinical utility of our SDH activity models, which may be used in conjunction with computational approaches.

### Functional stratification and ACMG/AMP evidence for SDHB VUS alleles.

Our primary goal was to develop a functional assay for evaluating *SDHB* VUS alleles that could be used to improve risk classification for autosomal dominant hPPGL. Toward this objective, we applied the activity-based logistic regression model, using *P(path)* and OddsPath values to assess missense variants lacking sufficient evidence for a definitive benign/likely benign (B/LB) or pathogenic/likely pathogenic (P/LP) classification. As a real-world test, we queried the Stanford Cancer Genetics Database (REDCap), which contained germline sequencing data from approximately 18,700 patients evaluated through the Stanford Cancer Genetics Program. From this cohort, we identified 17 *SDHB* missense variants reported as VUS at the time of testing. Additionally, we curated 10 VUS alleles from ClinVar to represent a broader spectrum of uncertain missense variants. Finally, we included 3 VUS alleles that are causative for recessive mitochondrial disease (Leigh syndrome, VUS^Leigh^) in the homozygous state, to examine whether their functional profiles differ from those of dominantly inherited PPGL-associated variants. Given that Leigh syndrome may be caused by hypomorphic alleles in the homozygous state, we predicted that these variants would offer a biologically distinct comparison group for assessing SDH functional thresholds.

**Figure 4:**
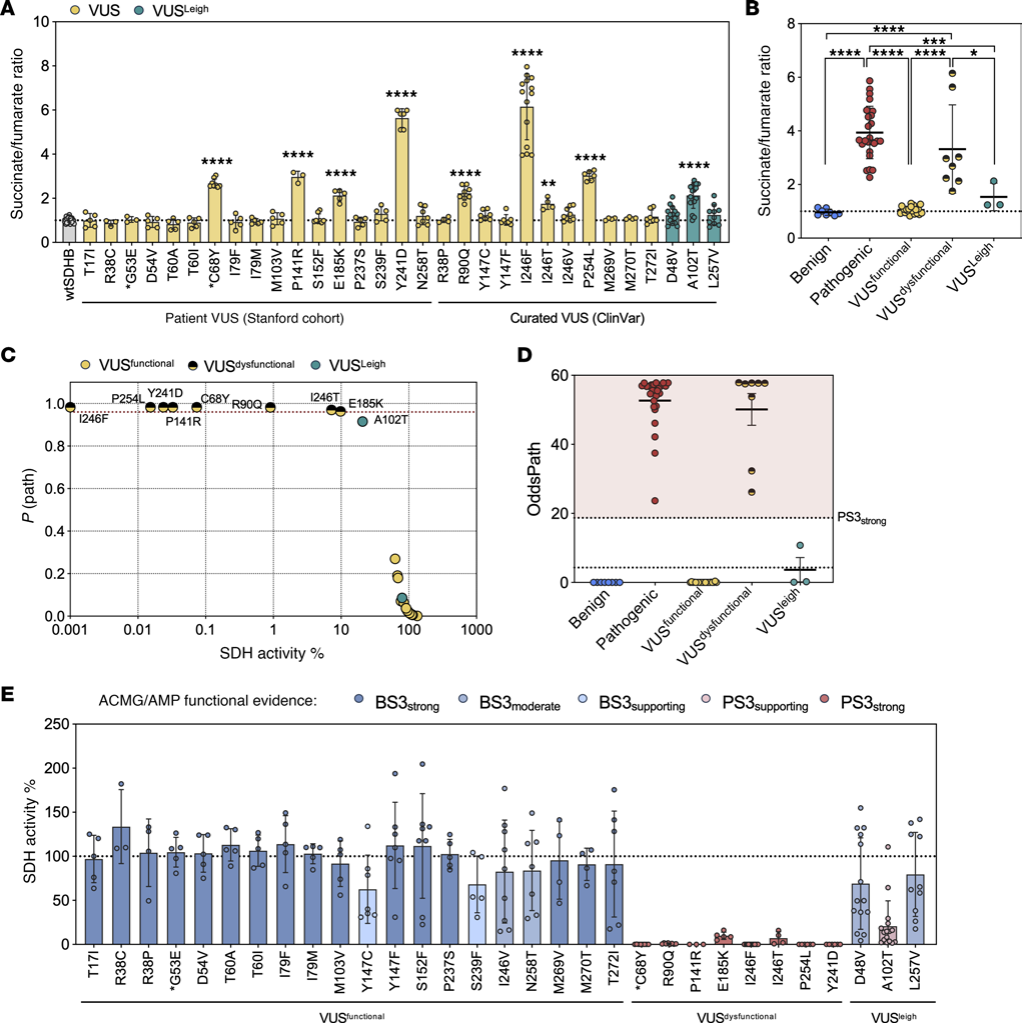
Functional characterization of SDHB VUS alleles enables ACMG/AMP evidence assignment for risk classification. (**A**) Succinate/fumarate ratios for *SDHB* variants of uncertain significance (VUS), including variants from Stanford patient database and ClinVar reports. VUS associated with Leigh Syndrome (VUS^Leigh^) presented in turquoise. Asterisk highlights the VUS that were recently reclassified. (**B**) Grouped comparison of succinate/fumarate ratios among known variants, VUS^Leigh^, and VUS categorized as functional or dysfunctional. (**C**) *P(path)* plots for VUS using the activity-based logistic regression model. Threshold of 0.95 represented by red dotted line. (**D**) Grouped comparison of OddsPath derived from modeled SDH activity. (**E**) SDH activity percentage and associated ACMG/AMP functional evidence that is color coded by strength. Each point represents a biological replicate. Each variant was tested in at least 2 independent experiments. Mean ± SD are shown. Ordinary 1-way ANOVA used in **A** (Dunnett’s multiple tests compared with wtSDHB) and **B** (Tukey’s multiple comparison test), ****P < 0.0001; ***P < 0.001; **P < 0.01; *P < 0.05.

Among the 31 VUS tested, 9 variants exhibited succinate/fumarate ratios significantly elevated relative to wtSDHB (Figure 4A) and to the mean of all known benign variants (Figure 4B), placing them within the SDH dysfunctional range. These results indicated that a subset of VUS alleles exhibit biochemical profiles consistent with pathogenic variants. From the Stanford cohort, SDHB^C68Y^, SDHB^P141R^, SDHB^E185K^, and SDHB^Y241D^ demonstrated elevated ratios. Notably, the SDHB^P141R^ variant was identified in a patient who underwent cancer genetic testing following a breast cancer diagnosis. Subsequent abdominal imaging, performed during evaluation for postoperative urinary retention, incidentally revealed a large hypervascular retroperitoneal mass. Surgical excision confirmed an 8 cm PGL with loss of SDHB expression by IHC. These pathological findings strongly support the pathogenicity of the SDHB^P141R^ variant and are consistent with its biochemical profile in our assay. Moreover, SDHB^C68Y^ and SDHB^G53E^ (indicated with asterisks) were recently reclassified in ClinVar as pathogenic and benign, respectively (Figure 4A), aligning with the results of our functional assay.

Next, to highlight the functional heterogeneity among the VUS alleles, we provisionally grouped them into 3 categories: VUS^dysfunctional^ (*n* = 8), exhibiting elevated ratios indicative of SDH impairment; VUS^functional^ (*n* = 19), exhibiting ratios consistent with full or near-complete functional rescue; and VUS^Leigh^, which are known to be causative for Leigh Syndrome. The range of dysfunction among VUS was also evident, with SDHB^I246T^ and SDHB^I246F^ showing the lowest (1.75) and highest (6.15) succinate/fumarate ratios among the dysfunctional group, highlighting the assay’s resolution across degrees of enzymatic impairment. Interestingly, VUS^Leigh^ exhibited succinate/fumarate ratios that partially overlapped with benign variants (Figure 4, A and B). This is consistent with the clinical observation that Leigh syndrome results from biallelic hypomorphic variants that allow partial SDH function and survival into infancy or childhood, in contrast with complete SDHB loss, which is incompatible with life, as evidenced by the embryonic lethality of SDHB-null mice (28, 40, 41).

To assess the functional categorization of VUS alleles, we applied the activity-based logistic regression model to calculate *P(path)* and OddsPath values. All 8 VUS (including the reclassified SDHB^C68Y^) identified as dysfunctional by succinate/fumarate ratio exhibited *P(path)* values above the 0.96 threshold, supporting their classification as likely pathogenic (Figure 4C). OddsPath analysis provided additional resolution of evidence strength: each of these variants reached the threshold for PS3_strong_, while all remaining VUS with WT–like activity fell within the BS3_supporting_ or BS3_moderate_ range (Figure 4, D and E). These classifications were biologically consistent, as dysfunctional variants exhibited markedly reduced SDH activity (–0.1%), comparable with known pathogenic controls. Among the 3 Leigh syndrome–associated variants, SDHB^A102T^ exceeded the pathogenicity threshold and was assigned PS3_supporting_ evidence, consistent with partial functional disruption. In contrast, SDHB^D48V^ and SDHB^L257V^ retained near-normal SDH activity and were classified as BS3.

### Functional assessment of domain-specific SDHB variants.

**Figure 5:**
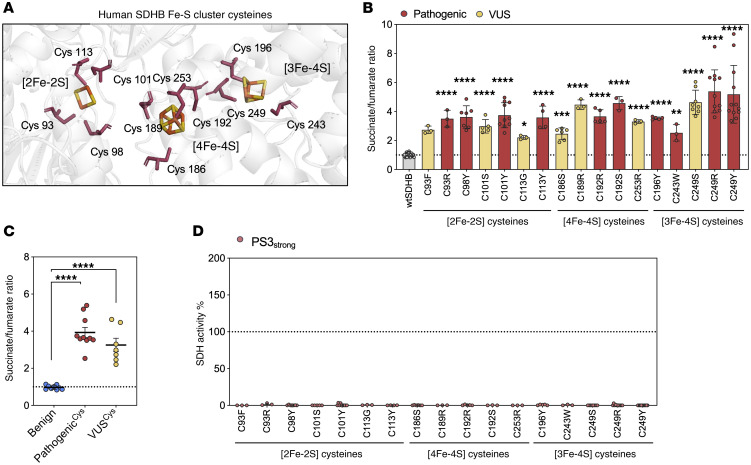
Conserved cysteine residues in SDHB are critical for SDH function and uniformly associated with pathogenicity. (**A**) Protein structure demonstrating the 11 cysteine residues associated with the 3 Fe-S clusters (2Fe-2S, 4Fe-4S and 3Fe-4S) in SDHB. Amino acid structure, pink; Fe atom, orange; S atom, yellow. Visualization created in PyMOL and shown in cartoon mode. (**B**) Succinate/fumarate ratio, (**C**) grouped comparisons to succinate/fumarate ratio and (**D**) SDH activity percentage in cysteine-associated VUS and known pathogenic variants. ACMG/AMP functional evidence is color coded by strength. Each point represents a biological replicate. Each variant was tested in at least 2 independent experiments. Mean ± SD is shown. Ordinary 1-way ANOVA used in **B**, **C** (Dunnett’s multiple tests compared with wtSDHB), ****P < 0.0001; **P < 0.01.

We next investigated whether mutations within specific domains of SDHB protein disrupt SDH enzymatic activity. First, we focused on the iron-sulfur (Fe-S) cluster–binding regions that are hypothesized to be essential for succinate dehydrogenase activity. SDHB contains 3 Fe-S clusters (2Fe–2S, 4Fe–4S, and 3Fe–4S), which are characterized by highly conserved cysteine residues that directly bind and stabilize iron atoms for efficient electron transfer (42). The 3D structure of human SDHB was examined to identify the spatial arrangement of all 11 Fe-S–coordinating cysteines (Figure 5A). We expected that coding substitutions of these residues would impair iron coordination and/or compromise SDHB structural integrity. Supporting this, known pathogenic variants affecting these cysteine residues exhibited markedly elevated succinate/fumarate ratios. We therefore hypothesized that VUS alleles impacting other conserved Fe-S cysteine residues (VUS^Cys^) would similarly disrupt SDH enzymatic function. Indeed, functional evaluation of all 7 VUS^Cys^ reported in ClinVar exhibited elevation of succinate/fumarate ratios relative to wtSDHB (Figure 5B) and benign variants (Figure 5C), consistent with loss of function. Based on OddsPath calculations and ACMG/AMP guidelines, all 7 variants met criteria for PS3_strong_ functional evidence classification (Figure 5D). These findings underscored the role of conserved cysteine residues in the Fe-S clusters of SDHB and support the pathogenicity of any variant that alters these residues.

**Figure 6:**
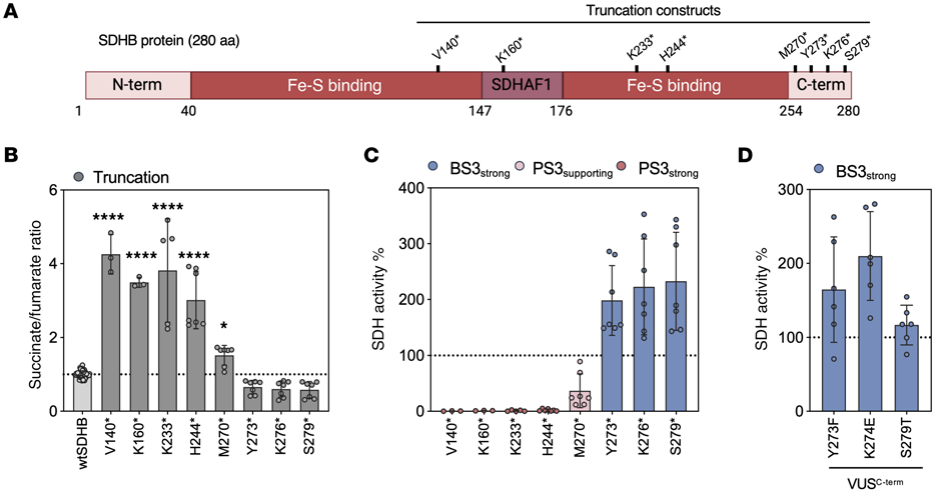
Truncation mapping defines essential functional domains of SDHB and supports variant interpretation framework. (**A**) Schematic summary of the domains of SDHB (N- and C-terminal, Fe-S binding, SDHAF1 binding region). Truncation constructs are indicated. (**B**) Succinate/fumarate ratio and (**C**) SDH activity percentage characterization in SDHB^KO^ cells transfected with truncation constructs. (**D**) SDH activity percentage of VUS found in the C-terminal domain (VUS^C-term^). ACMG/AMP functional evidence is color coded by strength. Each point represents a biological replicate. Each variant was tested in at least 2 independent experiments. Mean ± SD is shown. Ordinary 1-way ANOVA used in **B** and **C** (Dunnett’s multiple tests compared with wtSDHB), ****P < 0.0001; **P < 0.01.

Next, we tested successive C-terminal SDHB truncations, targeting key structural regions (including the Fe-S cluster–binding and SDHAF1-binding domains), to determine the “minimal” functional protein (Figure 6A). Notably, the C-terminal tail, which lacks defined structural or interaction motifs, harbors over 20 reported VUS alleles. These truncation constructs allowed us to evaluate whether the C-terminal aspect of SDHB was dispensable and would likely tolerate missense mutations. Each truncation construct introduced a premature stop codon at defined amino acid positions, thereby eliminating downstream residues while preserving upstream protein structure.

Constructs truncated at or before His244 failed to rescue succinate/fumarate levels and exhibited minimal SDH activity, consistent with complete loss of function and classification as PS3_strong_ (Figure 6, B and C). Met270*, which truncates just prior to the unstructured tail, showed intermediate activity and met criteria for PS3_supporting_. In contrast, C-terminal truncations at and beyond Tyr273 (Tyr273*, Lys276*, Ser279*) fully restored SDH activity and succinate/fumarate ratios, each meeting BS3_strong_ classification. These results delineate a functionally essential boundary within SDHB and support the dispensability of the distal C-terminal region for enzymatic activity. To validate this finding, we tested 3 VUS alleles located within the C-terminal tail — SDHB^Y273F^, SDHB^K274E^ and SDHB^S279T^ — all of which exhibited WT-like functional rescue and are therefore, likely benign/BS3 (Figure 6D). The expression of experimentally tested truncations and C-terminal variants was confirmed by Western blot (Supplemental Figure 3). All truncations at or beyond Lys233* were detected, whereas Val140* and Lys160* were not observed, which may reflect either reduced stability or lack of antibody recognition.

To demonstrate the clinical utility of our functional data for interpreting *SDHB* VUS alleles, we conducted a mock variant classification using the points-based framework recommended by ClinGen Variant curation expert panels (VCEP) (36). Points were assigned based on the strength of evidence from population frequency, computational predictions, and our functional assay, with the cumulative score determining the final pathogenicity classification (43). We compared classifications made without and with functional evidence, referred to as pre- and postfunctional classifications, respectively, for 39 VUS alleles (excluding SDHB^C68Y^ and SDHB^G53E^ which were reclassified in ClinVar) tested in this study (Table 1). Using only population and computational data (preclassification), 5 of the 39 VUS — SDHB^D48V^, SDHB^M103V^, SDHB^Y147C^, SDHB^S152F^, and SDHB^P237S^ — were classified as Likely Benign (LB). These interpretations were retained with the addition of functional evidence, but with much stronger support (final scores less than –6).

Among the 3 Leigh-associated VUS alleles, SDHB^D48V^ was classified as LB in both pre- and postfunctional classification; by contrast, SDHB^L257V^ and SDHB^A102T^ were classified as VUS alleles in both pre- and postfunctional classification. Interestingly, based upon functional testing, SDHB^L257V^ received BS3_moderate_ evidence while SDHB^A102T^ received PS3_moderate_ evidence. Thus, despite moderate functional evidence, the final scores for these 2 Leigh-associated alleles were insufficient for reclassification, indicating incomplete resolution — with respect to hPPGL risk — of Leigh-associated variants using this assay. Interestingly, computational prediction (REVEL) assigned PP3 to almost all VUS alleles, with scores ranging from supporting to strong. However, multiple variants predicted to be damaging in silico — such as SDHB^R38P^, SDHB^T60I^, and SDHB^S152F^—were reclassified as LB with functional testing. Thus, REVEL appeared to overpredict allele pathogenicity and underpredict allele benignity, emphasizing the added value of functional interrogation.

**Figure 7:**
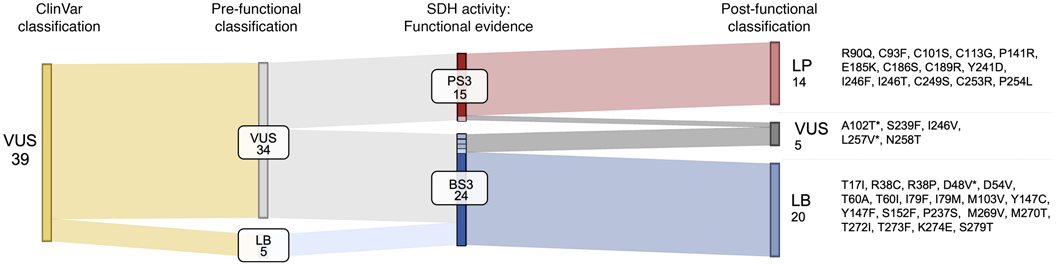
Clinical mock interpretation of SDHB VUS. Sankey diagram summarizing the results of mock variant interpretation prior to and after the addition of functional evidence. Colors in the functional evidence column correspond to the strength of evidence. Variants that are reclassified are listed in the ‘Post-functional Classification’ column. Asterisk indicates the Leigh syndrome-associated SDHB alleles.

All 14 variants that received functional PS3_strong_ evidence were reclassified as Likely Pathogenic (LP) with incorporation of functional assay data. This group included all cysteine substitutions of the SDHB iron-sulfur domain, reinforcing the functional indispensability of these residues. Notably, 7 variants — SDHB^C93F^, SDHB^C189R^, SDHB^Y241D^, SDHB^I246F^, SDHB^C249S^, SDHB^C253R^ and SDHB^P254L^ — achieved pathogenicity scores of 9, just 1 point below the threshold for full ‘Pathogenic’ classification (≥ 10). Overall, our functional data supported reclassification of 87% of VUS alleles (34 of 39), many of which were upgraded to a clinically actionable category (LP: 14 of 39), demonstrating immediate translational impact of this functional assay (Figure 7).

### Succinate/fumarate ratio predicts PPGL location likelihood for pathogenic SDHB Alleles.

**Figure 8:**
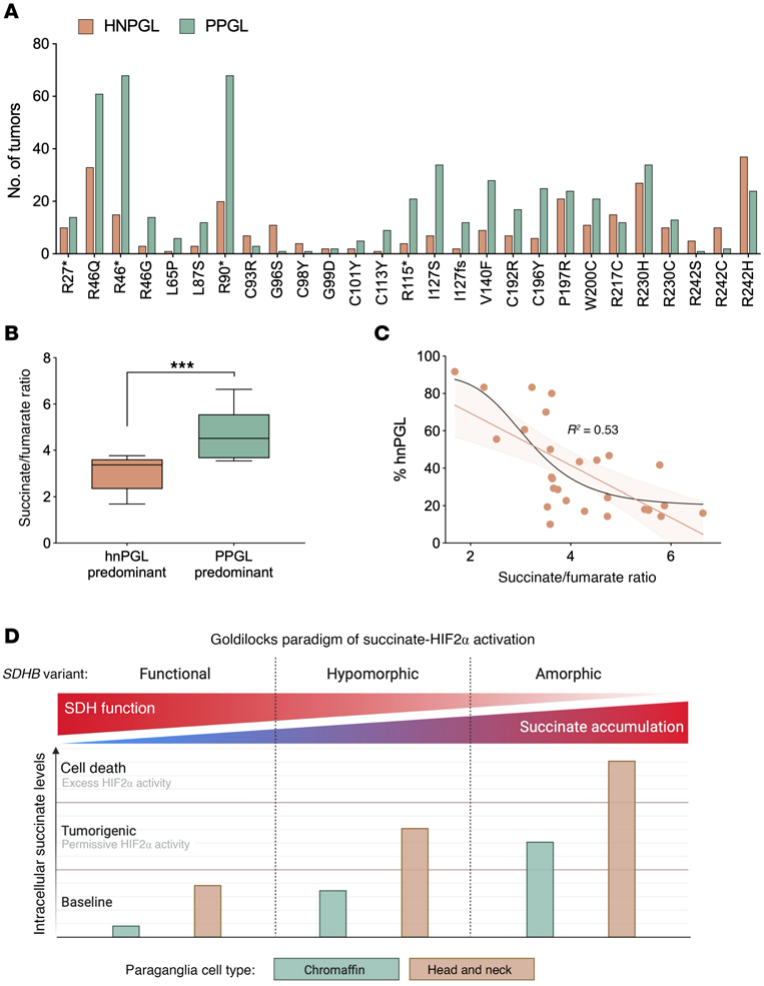
Succinate/fumarate ratios predict tumor location and distinguish *SDHB* variants associated with hnPGL from those linked to PPGL. (**A**) Number of hnPGL or PPGL tumors reported for each variant. (**B**) Grouped comparison of succinate/fumarate ratios in variants predominantly hnPGL (3.03 ± 0.77) or PPGL (4.63 ± 0.97). Predominance set at greater than 50%. (**C**) Correlation plots of succinate/fumarate ratio to the percentage of total tumors reported as hnPGL (% hnPGL). Linear regression model, orange line. Non-linear sigmodal mode, black line (R^2^ shown). (**D**) Schematic representation of our hypothetical model linking *SDHB* variant severity to tumor location. *SDHB* variants linked to hnPGL exhibited significantly lower succinate accumulation compared with those associated with PPGL. In our model, this gradient in succinate level drives differential HIF2-α stabilization, which we describe as the ‘Goldilocks paradigm of succinate-HIF2-α activation’. Created with BioRender.com. Unpaired Student’s *t* test used in **B**, ****P < 0.0001.

*SDHB* variants exhibit heterogenous expressivity, with some alleles more frequently associated with head and neck paraganglioma (hnPGL) and others with PPGL. Prior work showed that severely damaging *SDHB* truncating alleles were associated with increased risk of PPGL, while missense variants exhibited a nonsignificant trend toward increased risk of hnPGL (44). Accordingly, we hypothesized that a subset of less severe *SDHB* missense alleles, quantified by succinate/fumarate ratio, would correlate with increased hnPGL occurrence. Tumor distribution data for each variant were compiled from published clinical cases (Figure 8A and Supplemental Table 1). For each variant, hnPGL- or PPGL-predominant status was assigned based on whether greater than or equal to 50% of reported tumors occurred in that site. In our assay, variants classified as hnPGL-predominant exhibited significantly lower succinate/fumarate ratios than PPGL-predominant variants (Figure 8B and Supplemental Figure 4), suggesting that severely hypomorphic *SDHB* alleles may preferentially predispose individuals to hnPGL tumors, compared with amorphic *SDHB* alleles.

To evaluate the relationship between the succinate/fumarate ratio and the proportion of tumors classified as hnPGL, we first applied a simple linear model, which revealed a moderate correlation (R² = 0.452, *P* < 0.0001) (Figure 8C). However, given the biological expectation that hnPGL frequency may plateau at lower ratio values and diminish with higher ratios, we additionally fit a sigmoidal 4-parameter model to the same data. Overlaying both models demonstrated that the sigmoidal curve better captured the apparent trend (R² = 0.533, *P* = 0.0016). Overall, a significant inverse correlation was observed between the succinate/fumarate ratio and the percentage of tumors presenting as hnPGL. Variants with more severe biochemical disruption — reflected in higher succinate/fumarate ratios — were more commonly associated with PPGL. Our results align with the proposed succinate threshold model (45), in which differences in baseline succinate levels between precursor cell types shape the tumorigenic potential of *SDHB* variants (Figure 8D). Parasympathetic paraganglia, which give rise to hnPGL, are thought to have higher endogenous succinate levels and may therefore be more susceptible to additional succinate accumulation caused by hypomorphic *SDHB* mutations. In contrast, sympathetic paraganglia cells may require more severe SDH dysfunction to reach the threshold needed to trigger oncometabolite-driven tumorigenesis. The observation that *SDHB* variants associated with hnPGL exhibit lower succinate/fumarate ratios supports this model, and we propose that succinate levels may directly modulate the degree of HIF2-α stabilization (Figure 8D). Taken together, our results highlight a genotype-phenotype correlation model in which the degree of SDHB dysfunction contributes to location-specific tumorigenic risks.

## Discussion

The preponderance of *SDHB* missense alleles are categorized as nonactionable VUS, posing a critical barrier to appropriate tumor surveillance and familial testing or clinical reassurance. A robust functional assay to enhance definitive variant classification is needed. Herein, we present the most extensive functional characterization of *SDHB* missense variants to date (testing 75 alleles, including 9 benign, 27 pathogenic, and 39 VUS alleles) and validate a scalable, allele-specific complementation assay. Our biochemical approach reliably distinguished pathogenic from benign variants based on the succinate/fumarate ratio and SDH enzymatic activity. Using logistic regression modeling, we defined quantitative thresholds to assign ACMG/AMP functional evidence (PS3/BS3), supporting clinical variant interpretation in a structured, evidence-based manner. Notably, this assay resolved 87% of tested VUS alleles — classifying 16 as likely benign and 14 as likely pathogenic. Collectively, this work established a clinically actionable framework for assessing *SDHB* missense variants and advancing personalized care in *SDHB* hereditary paraganglioma and pheochromocytoma.

The need for a functional assay to resolve the pathogenicity of *SDHB* missense variants is similarly evident for other SDH subunits. For example, elegant recent work by Heinrich and colleagues used a conceptually similar complementation assay in SDHA-deficient cells to enhance *SDHA* variant interpretation (37). Their approach achieved comparably robust variant classification but involved greater technical complexity. Specifically, they employed a single copy, targeted knock-in strategy with coexpression of a GFP reporter to enable accurate assessment of SDHA variant protein stability (37). However, incorporating SDHA stability measurements into pathogenicity modeling had limited added benefit and was essentially dispensable. In contrast, our streamlined approach — transient transfection of *SDHB* variants followed by LC-MS/MS measurement of succinate and fumarate levels — offers a similarly robust functional classification with improved scalability and technical simplicity. Interestingly, our findings of SDH enzyme dysfunction, even in the setting of variant overexpression, casts doubt on the benefit of therapeutic approaches aimed at stabilizing pathogenic *SDHB* alleles — such as preventing proteasomal degradation. However, it remains possible that a subset of variants, including *SDHB* Leigh-associated alleles or hypomorphic PPGL-associated mutations, like SDHB^R242C/S^ (46), might benefit from targeted protein stabilization strategies.

Importantly, our study adhered to ACMG/AMP PS3 and BS3 guidelines, incorporating both benign and pathogenic controls to sufficiently demonstrate the interpretive value of the assay for clinical application. We note a variety of previous studies developed model systems to investigate *SDHB* allele function (46–49); however, these approaches were insufficiently pursued to enable clinical use. For example, yeast complementation assays, although limited by cross-species differences in protein folding and metabolic context, have been used to model *SDHB* variant dysfunction (46). Additionally, prior SDHB complementation studies were performed with SDHB-deficient UOK renal cell carcinoma cells and HEK293 using a limited number of pathogenic *SDHB* alleles (47, 49). Notably, we chose HEK293 cells for assay development because they are (a) highly transfectable, (b) derived from an hPPGL-affected lineage (kidney cells), and (c) viable in the setting of SDHB deficiency. We also generated SDHB-KO HeLa cells and confirmed successful complementation (data not shown); further supporting the broad adaptability of this assay to different cellular contexts. Notably, recent work by Gebhardt et al. reported differences in Krebs cycle metabolites, including succinate/fumarate ratios in plasma and red blood cells of presymptomatic patients with hPPGL harboring pathogenic *SDHx* variants (48). While this approach holds promise for stratifying *SDHx* alleles, it remains in the early stages of development and may lack the resolution necessary for clinical implementation (50). Thus, a major contribution of the current work is the extensive effort made to deliver a validated and scalable platform for assessing *SDHB* variant function that can be clinically applied.

Using the ACMG/AMP framework, our functional data provided strong evidence to reclassify several *SDHB* missense VUS alleles as likely pathogenic, particularly those affecting cysteine residues critical for coordinating Fe-S clusters—a structural feature essential for SDH enzymatic activity. These findings are consistent with prior studies in UOK269 cells, where pathogenic SDHB cysteine mutations disrupted succinate-coenzyme Q reductase (SQR) activity and impaired SDH complex assembly (47). However, our study is, to our knowledge, the first to functionally characterize and reclassify multiple VUS at Fe-S cluster-binding cysteines — including SDHB^C93F^, SDHB^C101S^, SDHB^C113G^, SDHB^C186S^, SDHB^C189R^, SDHB^C249S^, and SDHB^C253R^ — as likely pathogenic, based on PS3_strong_ evidence. Beyond canonical cysteine residues, we also categorized variants anticipated to indirectly disrupt Fe-S coordination through structural perturbation. One such variant, SDHB^P254L^, exhibited marked loss of function in our complementation assay and fulfilled criteria for likely pathogenicity. Structural modeling suggested that the proline-to-leucine substitution induces conformational changes that destabilize neighboring residues, including C253, a canonical Fe-S residue; this structural incompatibility likely compromises 4Fe-4S cluster binding (51). Importantly, our classification of SDHB^P254L^ as likely pathogenic is supported by the Next Generation Sequencing in Pheochromocytomas and Paragangliomas study group (NGSnPPGL) algorithm (52) and by clinical reports of this variant in individuals with hPPGL (53, 54). Together, these findings underscore the essential role of Fe-S cluster integrity in SDHB function and demonstrate how biochemical complementation can refine variant classification for improved clinical interpretation.

The integration of functional evidence helps clarify the pathogenicity of *SDHB* variants in clinical contexts where phenotypic expression is incomplete or delayed. For example, several patients from the Stanford Cancer Genetics Program carried variants — SDHB^C68Y^, SDHB^P141R^, SDHB^E185K^, and SDHB^Y241D^ — classified as likely pathogenic in our mock clinical interpretation; however, only the individual with the SDHB^P141R^ variant was known to have SDHB-related disease. The SDHB^C68Y^ variant, recently reclassified as likely pathogenic in ClinVar, was identified in a patient following a diagnosis of fallopian cancer; subsequent evaluation, including plasma metanephrines and full-body MRI at age 66 were normal. Similarly, the SDHB^Y241D^ variant was identified in a patient evaluated after a breast cancer diagnosis at age 68, with normal plasma metanephrines and full body MRI. While the SDHB^Y241D^ variant remains a VUS in ClinVar, it was previously reported in a case of metastatic PGL (55). Another patient carrying the SDHB^E185K^ variant, identified as a result of a familial glioblastoma history, has not undergone relevant imaging or biochemical screening to date. These cases highlight the incomplete penetrance of pathogenic *SDHB* mutations, estimated at approximately 20%–30%, and underscore the challenges of clinical interpretation in the absence of definitive disease manifestations. Incorporating functional data into variant classification frameworks may therefore provide valuable guidance for risk assessment and management of such individuals.

*SDHx* variant pathogenicity should be interpreted within disease-specific contexts (3, 37). While *SDHx* variants underlie both dominantly inherited PPGL and recessively inherited primary mitochondrial disease (PMD), our data (and prior evidence) indicate that these conditions are caused by distinct variants (56). To our knowledge, PPGLs have not been observed in individuals who are heterozygous for an *SDHB* allele that causes PMD in the homozygous state. In our assay, 2 out of 3 *SDHB* variants previously associated with Leigh syndrome in a homozygous state (SDHB^D48V^ and SDHB^L257V^) exhibited WT-like succinate/fumarate ratios and SDH activity levels. However, SDHB^A102T^ showed a modestly elevated succinate/fumarate ratio — approximately double that of WT — with approximately 20% SDH activity. This partial dysfunction may be explained by structural modeling, which suggests that the threonine substitution at position 102 introduces a nonnative polar interaction with Cys189 — an Fe-S cluster–coordinating residue — potentially disrupting local tertiary structure, impairing cluster incorporation, and destabilizing complex II assembly (57). Interestingly, Kent et al. reported a similar functional overlap between SDHA-associated PMD variants and benign alleles (37), suggesting that the retained SDH activity of Leigh Syndrome variants in our assay is not simply an artifact of overexpression. These shared findings raise the possibility that the pathogenicity of *SDHA/B* PMD alleles is not solely attributable to impaired succinate oxidation but reflects a distinct pathogenic mechanism, such as altered ROS generation (58–60). Altogether, our data support the notion that *SDHB* variants, which are compatible with life in the homozygous state, are unlikely to confer PPGL risk, highlighting the need for disease-specific frameworks to interpret *SDHx* variant pathogenicity; specifically, *SDHB* allele disease risk classification should be separately stated for hPPGL and PMD.

Our study provides interesting new insight into the relationship between *SDHB* variant metabolic function and tumor location, distinguishing hnPGL- and PPGL-predominant missense alleles. Prior work by Bayley et al. showed that truncating *SDHB* variants confer a higher risk for both PPGL and malignant transformation relative to missense variants (44). Expanding on this concept, we found that allele function (succinate/fumarate ratio) may yield variant-specific tumor risk profiles. We observed that *SDHB* variants predominantly associated with hnPGL exhibited lower succinate/fumarate ratios compared with PPGL-associated variants. For example, SDHB^R242C^ displayed a moderate succinate/fumarate ratio and is predominantly (83%) hnPGL-associated (61–68). This is consistent with prior yeast-based functional studies, which also categorized SDHB^R242C^ as a ‘mildly’ impaired variant (46). Importantly, our observation that hnPGL-associated variants accumulate less succinate is supported by tumor-based metabolomic studies, which reported lower succinate/fumarate ratios in hnPGL compared with PPGL tumors (27). While the lower succinate/fumarate ratios in hnPGL tumors was primarily driven by elevated fumarate in hnPGL tumors, our complementation assay revealed reduced succinate accumulation as the key determinant of this metabolic distinction. Together, these data provide a functional basis for a genotype-phenotype correlation, in which the extent of SDH activity loss influences tumor site predilection.

Several mechanistic models have been proposed to explain why the severity of SDHB dysfunction influences tumor location. Bayley and DeVilee outlined a 2-part model to explain why less severe *SDHB* missense mutations, compared with more disruptive truncating variants, are more likely to cause hnPGL (45). First, they proposed that mildly dysfunctional *SDHB* alleles increase ROS production and hypothesized that sympathetic chromaffin cells (PPGL precursors) are more sensitive to ROS toxicity, leading to cell death, whereas parasympathetic paraganglia cells (hnPGL precursors), specialized in oxygen sensing, are more ROS tolerant. Second, they introduced a succinate threshold concept: head and neck paraganglia, with higher baseline succinate are more susceptible to additional succinate accumulation. This second concept aligns with the oncometabolite hypothesis, in which excess succinate inhibits α-ketoglutarate–dependent enzymes such as prolyl hydroxylases and histone/DNA demethylases (TET and JmjC families), leading to HIF-α stabilization, pseudohypoxic signaling, and widespread epigenetic reprogramming (28, 69). While our data do not directly test this model, our observation that *SDHB* variants with lower succinate/fumarate ratios are predominantly associated with hnPGL supports its plausibility. A mechanistic parallel is seen in von Hippel–Lindau–associated (VHL-associated) tumors. Abu-Remaileh et al. showed that moderate HIF2-α stabilization promotes PPGL formation whereas excessive accumulation is cytotoxic, supporting a “Goldilocks” model of tumorigenesis (70). Applying this framework to SDHB, we propose that hypomorphic *SDHB* mutations yield intermediate HIF2α stabilization, sufficient for hnPGL where basal HIF2α expression is higher, while severe SDHB dysfunction causes greater succinate buildup and excessive HIF2-α stabilization, favoring PPGL (Figure 8D). Supporting our hypothesis, in vitro disruption of SDHB in chromaffin cells more robustly induced HIF2-α protein levels than SDHD disruption (71), potentially explaining the predominance of hnPGL with *SDHD* mutations compared with *SDHB* mutations. Further investigation is warranted to test our Goldilocks HIF2-α model of SDHB tumorigenesis and refine genotype-phenotype correlations across *SDHx* variants. Notably, a prediction of our model is that HIF2-α inhibition, for example, with Belzutifan, could have context-dependent therapeutic and tumor-promoting activities.

This study has noteworthy limitations. Our functional assay is designed to quantify biochemical disruption of SDH activity; however, it is not readily applicable to all mechanisms of allele disruption, including noncoding or intronic variants, splicing defects, dominant-negative effects, or alterations in transcriptional or epigenetic regulation. Additionally, use of transient transfection and artificial promoters results in nonphysiologic overexpression, which may obscure subtle loss-of-function effects seen in hypomorphic alleles. Additionally, enzyme complementation assays are intrinsically susceptible to false-positive pathogenic calls, i.e., functional alleles can be categorized as nonfunction on the basis of failed experimental execution. Hence, it is critical that purported loss-of-function alleles are independently tested multiple times to ensure accurate interpretation and to account for potential variability in the metabolic state of SDHB-KO cells. Experimentally, we observed that hypomorphic alleles and Leigh-associated variants demonstrated the highest degree of variability in our assay; however, repeated experimentation yielded clear delineation between dysfunctional and functional variants, with the potential exception of PMD-associated *SDHB* A102T. While succinate/fumarate ratio and SDH activity reliably predicts PPGL-associated *SDHB* alleles, these biochemical readouts are not appropriate for identification of PMD-associated *SDHB* alleles. Finally, although the genotype-phenotype correlation we uncovered is statistically robust, the finding is drawn from a relatively small patient and variant sample size. Hence, expanded and independent experimentation is warranted.

## Methods

### Sex as a biological variable.

This study does not involve human or animal subjects. Sex was not considered as a biological variable in selection of *SDHB* variants. *SDHB* variants were selected based on clinical occurrence and availability in database.

### Cell culture.

HEK293 (ATCC; RRID:CVCL_0045) and HEK293T (ATCC; RRID:CVCL_0063) cells were used for generation of SDHB-KO model and lentiviral transduction, respectively. All cells were cultured in DMEM/high glucose supplemented with 10% FBS and 1% Pen/Strep and maintained in a humidified incubator at 37°C, 5% CO_2_. All experiments were performed using SDHB-KO cells at passages 4–20. Mycoplasma contamination was frequently tested using e-Myco PLUS Mycoplasma PCR Detection Kit (Bulldog Bio; #25234).

### Generation of SDHB-knockout cell line.

Clonal SDHB-KO cell lines were generated by lentiviral transduction followed by antibiotic selection and monoclonal expansion. To produce single-guide RNA sequence (sgRNA) lentivirus, either pMCB320 (RRID:Addgene_89359) containing SDHB sgRNA #1 targeting exon 1 (TTGTTGGCGGAGCCTGCCTGC) or pLV[CRISPR]-hCas9 (Vector Builder) containing SDHB sgRNA #2 targeting exon 3 (ACACTCTAGCTTGCACCCGA) was combined with 3rd-generation lentiviral packaging mix (equal parts of pMD2.G (RRID:Addgene_12259), pRSV-Rev (RRID:Addgene_12253), and pMDLg/pRRE (RRID:Addgene_12251) at a 1:1 ratio (8 μg) and transfected into HEK293T cells using Lipofectamine 2000 according to manufacturer’s protocol (Thermo Scientific; L300015). 72 hr posttransfection, conditioned medium containing the viral particles was collected and filtered through a 0.45 μM Pore sterile filter (Corning). HEK-Cas9 cells (RRID:CVCL_UR28) or HEK293 were infected with virus media supplemented with 10 μg/mL polybrene. Infected cells were selected with 2 μg/mL puromycin for 5 days. Single clonal cell lines were generated by 96-well serial dilution method and functionally characterized before selection. Two SDHB-KO models were generated and used to rule out cell-cell variability and strengthen our functional analysis of *SDHB* variants. The comparable performance of the 2 SDHB-KO models is shown in Supplemental Figure 1. The results presented in this study were obtained using both models.

### Selection of known SDHB variants.

*SDHB* variants with known clinical classifications were obtained from ClinVar (RRID:SCR_006169) to establish assay performance characteristics. Variants were included if they had at least one ACMG (RRID:SCR_005769)-based classification of benign/likely benign (B/LB) or pathogenic/likely pathogenic (P/LP), and were used as benign or pathogenic controls, respectively (accessed 2023-2024). A total of 9 benign and 27 pathogenic variants were selected for model training and performance evaluation. Variants associated with hnPGL or PPGL, including truncating, frameshift, and missense types, were also collected from previous studies by Bayley et al. (44) and the number of tumors associated with each variant was recorded when available through literature search (accessed February 2025).

### Selection of SDHB VUS.

VUS alleles were selected based on either uncertain clinical evidence or conflicting interpretations in ClinVar (accessed 2023-2024). We queried the Stanford Cancer Genetics Database, a REDCap (RRID:SCR_003445)-based registry containing germline sequencing results from 18,633 individuals evaluated through the Stanford Cancer Genetics Program. Patients harboring any *SDHB* variant were identified, and cases with a reported VUS at the time of testing were included for functional assessment. Changes in clinical interpretation were documented to track any updates.

### Expression of SDHB variants.

*SDHB* variant constructs were designed on pTwist-CMV-BetaGlobin-WPRE-Neo (Twist Biosciences; RRID:SCR_025817). Homology sequence of human WT SDHB (wtSDHB) was designed by changing the Kozak sequence to “ACC” before the ATG start codon. SDHB-KO cells were plated at 250K cells/6-well and transfected with pTwist-*SDHB* variant plasmids using Lipofectamine 2000, according to the manufacturer’s protocol. Media was replenished after 4–6 h and cells cultured an additional 24–48 h. The minimum transfection concentration of 500 ng was determined by a wtSDHB dosing experiment (Supplemental Figure 2). To account for assay-to-assay variability, mock and wtSDHB transfections were performed with every experiment and served as the outcome metric for comparison.

### Succinate and Fumarate Measurements.

Targeted LC-MS analysis was performed to measure succinate and fumarate levels. Metabolites were extracted in 80% Ultra LC-MS–grade methanol on dry ice and centrifuged at 14,000 rpm for 15 min. Supernatant was collected for analysis using an Agilent 1290 UHPLC system coupled to an Agilent 6495C triple quadrupole mass spectrometer (LC-MS/MS). Analytes were detected in negative mode using the precursor to product transitions of 117–73.1 and 115–71.1 for succinate and fumarate, respectively. Peak quantification relative to the standard curve was performed using Agilent Masshunter Qualitative Analysis program (RRID:SCR_019081). An experimental run was excluded if functional rescue of wtSDHB transfection was less than 2-fold or more than 10-fold compared with SDHB-KO. All variants were tested in at least 2 independent experiments.

### Succinate/fumarate ratio and SDH activity calculations.

SDH enzymatic activity was calculated from measured succinate/fumarate ratios in cells expressing *SDHB* variants. For each independent experiment, mock- and WT-transfected SDHB-KO cells were included as control samples. SDH activity was modeled as an exponential function of Succinate/Fumarate ratio, constrained such that WT activity was set to 100% and KO activity to 0.1%. Specifically, SDH activity was calculated using the equation:
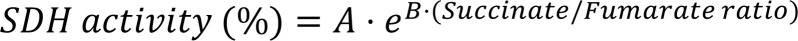


where constants A and B, respectively were determined for each experiment.
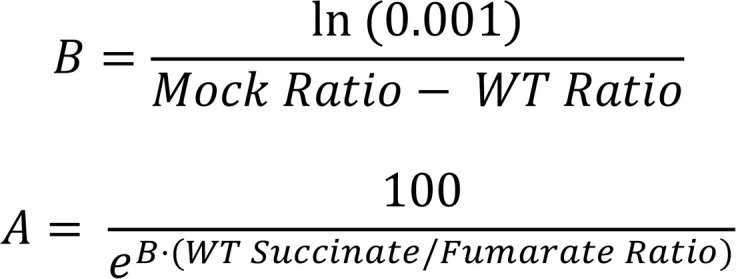


This approach enables consistent normalization across experiments with variable control ratios. The resulting percentage SDH activity values were used for pathogenicity modeling and functional classification.

### Complex II respiratory activity.

Complex II activity was assessed by measuring oxygen consumption rate (OCR) using a Seahorse XFe96 Analyzer (Agilent Technologies; RRID:SCR_019545), as previously described (72). Briefly, cells were seeded in 96-well Agilent Seahorse XF Cell Culture Microplate, incubated overnight at 37°C, 5% CO_2_ and permeabilized with 1 nM XF Plasma Membrane Permeabilizer (PMP) reagent diluted in mitochondrial assay solution (MAS) containing 4 mM ADP. OCR was measured in response to the following: port A, 2 μM rotenone; port B, 10 mM succinate and 4 uM rotenone; port C, 4 μM antimycin A. Data was presented as percentage of basal OCR.

### Western blotting.

Cell lysates were prepared using RIPA buffer supplemented with 1X protease and phosphatase inhibitor cocktail (Fisher Scientific). Proteins (20 μg) were prepared in Laemelli Buffer, denatured at 95°C for 5 min and separated on 10% or 12% gels by SDS-PAGE. Following electrophoresis, proteins were transferred onto PVDF membrane, blocked in Intercept (TBS) blocking buffer and incubated at 4°C overnight with the following primary antibodies: Total OXPHOS Cocktail (Abcam ab110413; 1:1000; RRID:AB_2629281), anti-SDHB (Abcam ab14714; 1:1000; RRID:AB_301432), anti-SDHA (Abcam ab14715; 1:1000), anti-β-actin (Sigma-Aldrich A5316; 1:5000; RRID:AB_476743), and anti-Actin (Abcam ab179467; 1:5000). Antibodies were detected with IRDyes Goat anti-Rabbit 800CW (LICORbio 925-3211; 1:20,000) or Goat anti-Mouse 680RD (LICORbio 925-68070; 1:20,000) and imaged on a LI-COR Odyssey CLX imaging system (LI-COR Biosciences; RRID:SCR_014579). Relative band signal intensity was quantified on Odyssey Image Studio Version 2.0 and normalized to loading control.

### Logistic regression modeling of variant pathogenicity and OddsPath calculations.

Logistic regression was used to compute the predicted probability of pathogenicity, *P(path)*, from the succinate/fumarate ratio directly or the calculated SDH enzymatic activity percentage. Models were implemented using the scikit-learn v1.6.1 library (RRID:SCR_002577). For each feature, a separate model was trained using a scikit-learn pipeline that included feature standardization (StandardScaler) and logistic regression with L1 regularization and the liblinear solver. To optimize model performance, the regularization strength (C) was selected using GridSearchCV. The cross-validation scheme employed 5 splits with 5 repetitions using RepeatedKFold, and models were evaluated based on minimization of log loss. We set class_weight = ‘balanced’ to account for class imbalance in the training dataset, ensuring equal penalization of misclassification across benign and pathogenic variants. Once optimal hyperparameters were selected, final models were retrained on the full known variant dataset. The threshold for functional classification was determined using Youden’s J statistic (73). Odds of pathogenicity (OddsPath) were derived from model probabilities using the formula: OddsPath = p/1-p. where p is the predicted probability of pathogenicity from the logistic regression model. This represents the odds that a given variant is pathogenic versus benign based on the logistic regression output. OddsPath values were subsequently used to assign ACMG-style functional evidence strengths, BS3 or PS3 (34).

### Comparison with computational model, REVEL.

REVEL scores for all *SDHB* variants were obtained from ClinGen Allele Registry (39). True positive rate (TPR) and positive predictive value (PPV) were calculated across thresholds from 0 to 1 for 3 predictors of pathogenicity: REVEL score, logistic regression model based on succinate/fumarate ratio, and model based on SDH activity. At each threshold, TPR was defined as the fraction of pathogenic variants correctly classified, and PPV as the fraction of variants classified as pathogenic that were truly pathogenic. TPR and PPV were also calculated using p(benignity) to assess benign classification performance. Established threshold of 0.644 (PP3) and 0.290 (BP4) were used for REVEL (38).

### Mock clinical variant interpretations.

SDHB variants were classified according to the points-based system developed by ClinGen Sequence Variant Interpretation Working Group (RRID:SCR_014968) (43). Classifications were made using population frequency data (BS1 and PM2), computational prediction scores (BP4 and PP3), and functional evidence (BS3 and PS3). Numerical values were assigned evidence strengths: supporting (1 point), moderate (2 points), strong (4 points), and very strong (8 points). Final classifications are based on cumulative scores, with thresholds as follows: pathogenic, with scores greater than or equal t0 10; likely pathogenic, with scores from 6–9; VUS, with scores form 0–5; likely benign, with scores from −6 to −1; and benign, with scores less than or equal to −7. For BS1 and PM2, we obtained population frequency of each variant from gnomAD v.4.1 (RRID:SCR_014964; accessed April 2025) and used a disease-specific thresholding strategy based on a dominant disease model for *SDHB*. Parameters included a disease prevalence of 1 in 3,000, an allelic contribution of 10%, and a penetrance estimate of 30% (74), resulting in a maximum credible population allele frequency (MCAF) of 5.55 × 10^–5^. Variants with a filtering allele frequency (FAF) greater than 1.39 × 10^–4^ (2.5-fold higher than MCAF) were assigned BS1_strong_, while those with FAF between 5.55 × 10^–5^ and 1.39 × 10^–4^ were assigned BS1_supporting_. Variants with a minor allele frequency (MAF) below 1.0 × 10^–5^ or absent from gnomAD were assigned PM2_supporting_ (37, 75). Computational evidence was derived from REVEL scores, and evidence strengths (BP4 or PP3) were assigned based on calibrated thresholds according to established criteria (38). Functional evidence was incorporated using OddsPath scores, which determined the appropriate PS3 or BS3 strength under ACMG/AMP guidelines.

### Statistics.

Statistical analyses were performed using GraphPad Prism v10 (RRID:SCR_002798). Data are presented as mean ± SD, and *P* < 0.05 was considered statistically significant. Comparisons between two groups were performed using 2-tailed unpaired Student’s t test. Comparisons among multiple groups were performed using ordinary 1-way ANOVA, followed by Tukey’s or Dunnett’s multiple-comparison post hoc tests. Details of sample size (*n*) and specific statistical tests are provided in the figure legends. For Figure 2, A and C, statistical analyses were conducted under the assumption of a lognormal distribution, as succinate/fumarate ratios and SDH activity measurements were positively skewed. In these analyses, group comparisons were performed using 1-way ANOVA with Dunnett’s multiple-comparison test, comparing each SDHB missense variant with wtSDHB.

### Study approval.

All research methods were approved by Stanford University’s Administrative Panel on Biosafety.

### Data availability.

Original data generated and analyzed during this study are included in this published article. Values for all data points in graphs are reported in the Supporting Data Values file.

## Author contributions

SL and JPA conceptualized the study, analyzed the data, interpreted experiments and wrote the manuscript. SL developed the biochemical assay. SL, LN and JP performed experiments. RCS performed LC-MS/MS analysis. SL and QG performed Western blot analysis. LN and JMF queried the Stanford Cancer Genetics Database. All authors approved the manuscript.

## Funding Support

This work is the result of NIH funding, in whole or in part, and is subject to the NIH Public Access Policy. Through acceptance of this federal funding, the NIH has been given a right to make the work publicly available in PubMed Central.

- NIH NIDDK (JPA: U01DK136965, R01DK101530 and R01DK119955; JPA and LN: T32DK007217).
- The Neuroendocrine Tumor Research Foundation (QG).
- A philanthropic gift from the Lau Family to advance research on SDHB-related disease.
- Research grants from the SDHB PheoPara Coalition (JPA).
- A philanthropic gift from the Lau Family to advance research on SDHB-related disease (JPA).
- Stanford Cancer Institute and the Breast Cancer Research Foundation (JMF).

## Supplementary Material

Supplemental data

Unedited blot and gel images

Supplemental table 1

Supporting data values

## Figures and Tables

**Table 1:**
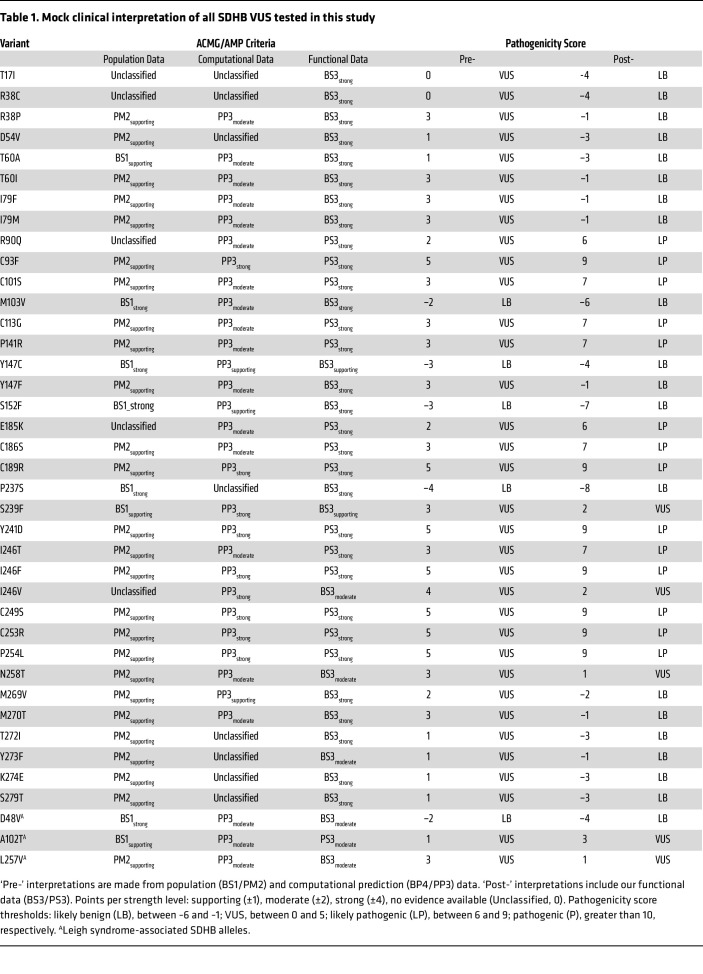
Mock clinical interpretation of all SDHB VUS tested in this study

## Version 1 11/18/2025

In-Press Preview

## Version 2 02/16/2026

Electronic publication
