# Supplementary material for: Functional characterization of SDHB variants clarifies hereditary pheochromocytoma and paraganglioma risk and genotype-phenotype relationships
Source: J Clin Invest. 2025 Nov 18;136(4):e198165. doi: 10.1172/JCI198165 (PMC12904712; doi:10.1172/JCI198165)
Supplement: Supplemental data [file jci-136-198165-s088.pdf]

Supplemental

Functional Characterization of SDHB Variants Clarifies Hereditary Pheochromocytoma and Paraganglioma Risk and Genotype–Phenotype Relationships

Sooyeon Lee[1], Leor Needleman[1], Julie Park[1], Rebecca C. Schugar[1], Qianjin Guo[1], James M. Ford[2], Justin P. Annes[1,3*]

[1]Department of Medicine, Division of Endocrinology, Stanford University, Stanford, CA

[2]Department of Medicine, Division of Oncology, Stanford University, Stanford, CA

[3]Stanford ChEM-H and Endocrine Oncology, Stanford University Cancer Institute, Stanford, CA

Corresponding Author

Justin P. Annes

CCSR 2255-A, 1291 Welch Rd., Stanford, CA 94305-5165

Phone: +1-650-736-2718

Email: jannes@stanford.edu

Supplemental Figures

Supplemental Figure 1. Generation of SDHB-KO cells.

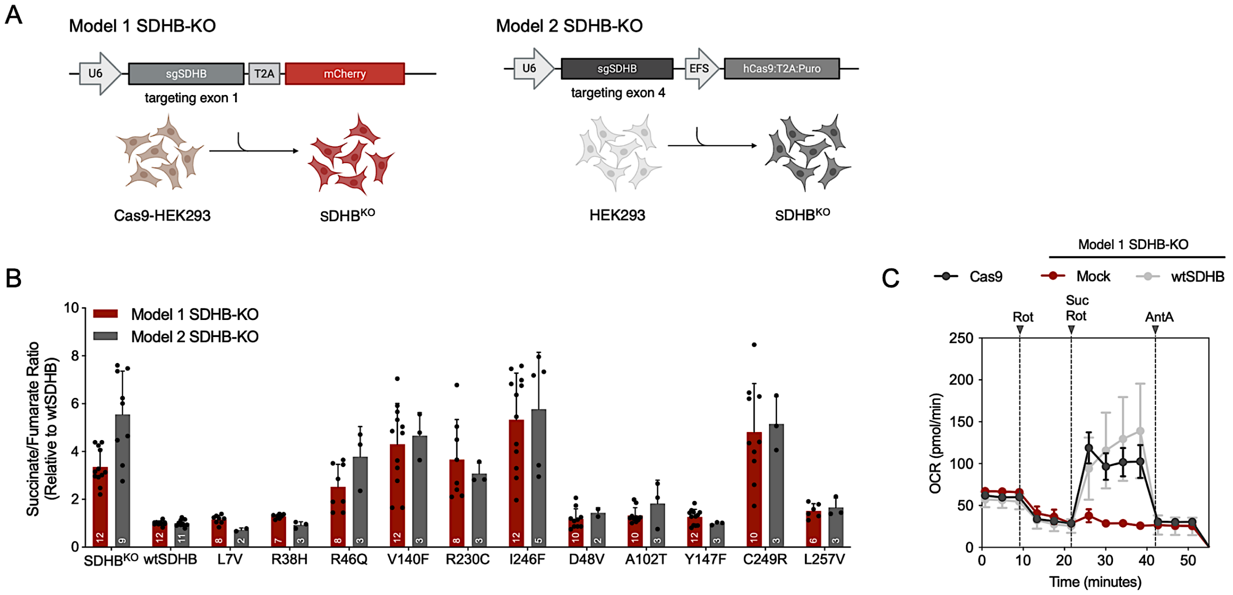

(**A**) Diagram of CRISPR/Cas9 vectors used to generate SDHB knockout model 1 and model 2. Model 1 construct is pMCB320 containing SDHB sgRNA #1 targeting exon 1. Model 2 is pLV[CRISPR]-hCas9 (Vector Builder) containing SDHB sgRNA #2 targeting exon 4. (**B**) Succinate/fumarate ratios of *SDHB* variants tested in model 1 and 2. (**C**) Oxygen consumption rate (OCR) assessing Complex II activity in Cas9-expressing HEK293 cells and mock- and wtSDHB-transfected SDHB-KO cells. Suc, Succinate. Rot, Rotenone. AntA, Antimycin A. Mean ± SD are shown.

Supplemental Figure 2. Confirmation of SDHB-KO cells.

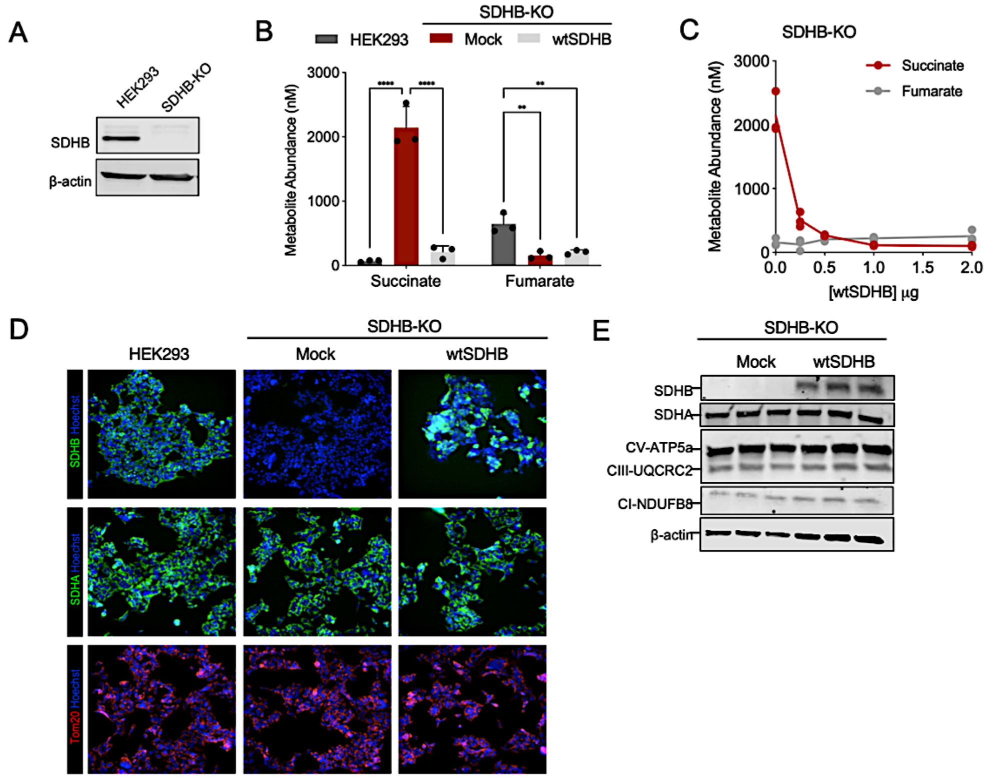

(**A**) Western blot of SDHB expression in lysates from HEK293 and SDHB-KO cells. (**B**) Succinate and fumarate levels in HEK293 and mock- and wtSDHB-transfected SDHB-KO cells. (**C**) Dosing effect of wtSDHB on succinate and fumarate levels. (**D**) Representative fluorescent staining of SDHB, SDHA (other SDH complex subunit) and Tom20 (mitochondrial marker). (**E**) Western blot of SDHB, SDHA and total OXPHOS, including Complex I, III and V. β-actin served as loading control for western blots. All data from model 2 SDHB-KO cells. Mean ± SD is shown. Ordinary two-way ANOVA used in B (Sidak's multiple comparisons test), ****$p < 0.0001$; **$p < 0.01$.

Supplemental Figure 3. Expression of *SDHB* variants in SDHB-KO cells.

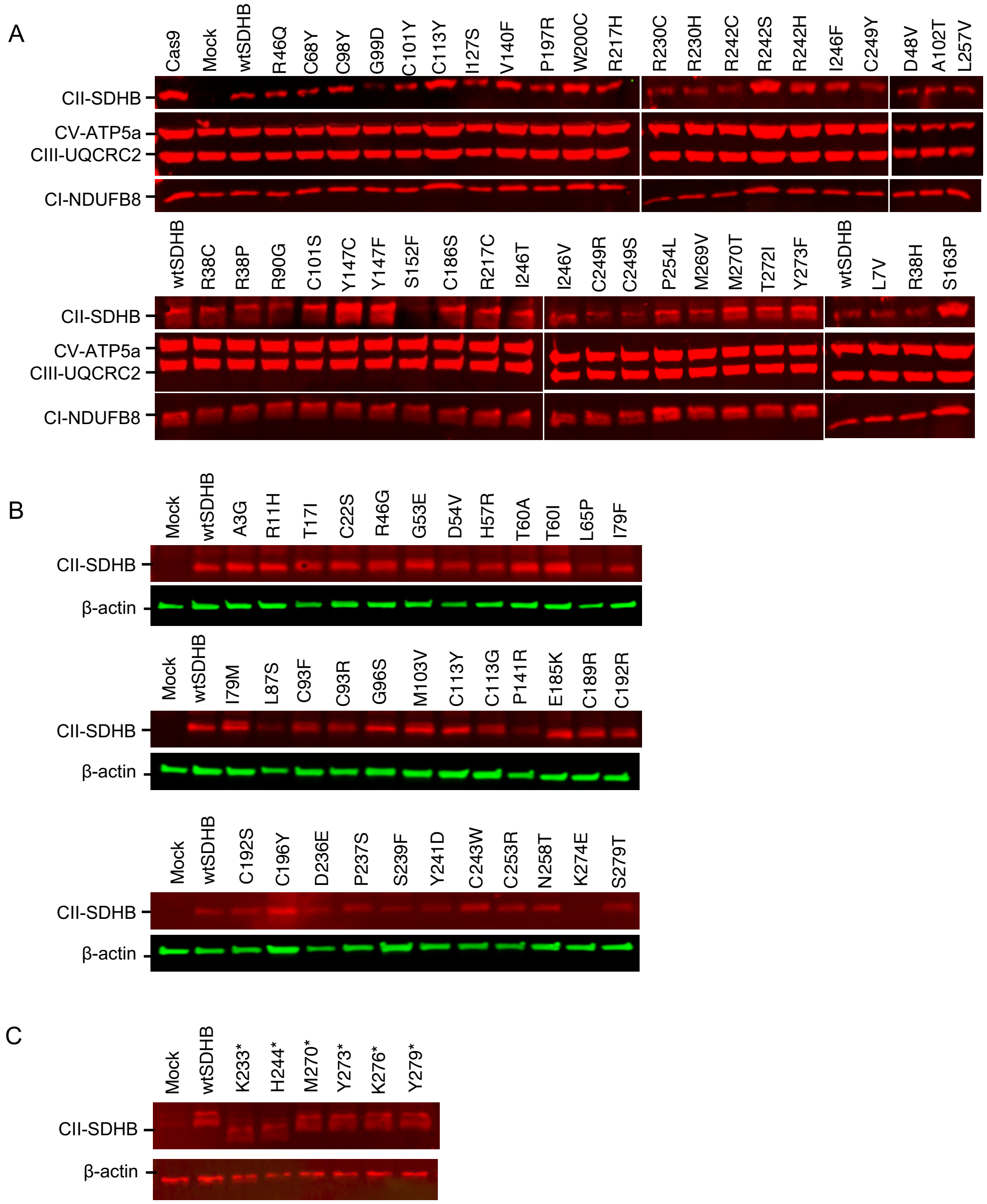

(**A-B**) Western blot of SDHB expression in lysates from HEK293 and SDHB-KO cells transfected with all variants tested in this study. (**A**) Mitochondrial protein CV-ATP5a, CIII-UQCRC2 and CI-NDUFB8 served as loading control. (**B**) β-actin served as loading control for western blots. (**C**) Truncation constructs of SDHB.

Supplemental Figure 4.

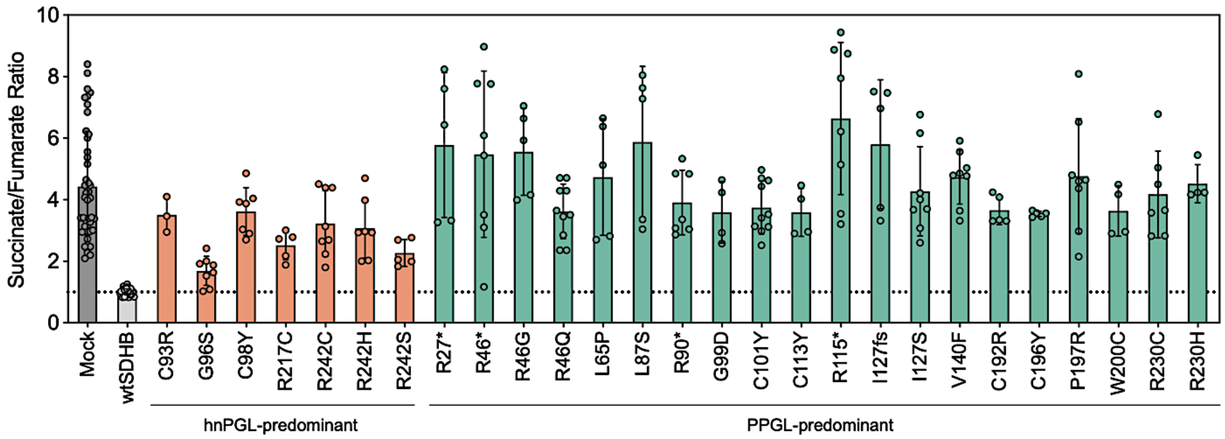

Succinate/fumarate ratios for SDHB variants with reported hnPGL or PPGL tumors. Each point represents a biological replicate. Each variant was tested in at least two independent experiments. Mean ± SD are shown.
