# Supplementary material for: Functional characterization of SDHB variants clarifies hereditary pheochromocytoma and paraganglioma risk and genotype-phenotype relationships
Source: J Clin Invest. 2025 Nov 18;136(4):e198165. doi: 10.1172/JCI198165 (PMC12904712; doi:10.1172/JCI198165)
Supplement: Unedited blot and gel images [file jci-136-198165-s089.pdf]

Full unedited blot/gel
All blots were imaged using Odyssey CLx (LI-COR Biosciences).
Molecular Weight Marker= MWM

Supplemental Figure 2A

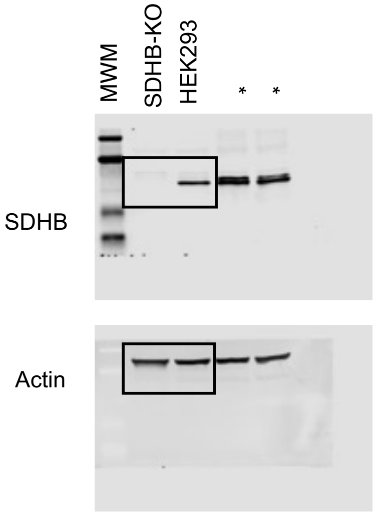

Anti-SDHB Antibody (abcam 14714) and anti-actin (abcam179467) was blotted on the same membrane. Image was flipped horizontally and cropped for final figure. Other lanes on blot (marked with *) were unrelated experimental samples for a different project

Supplemental Figure 2E

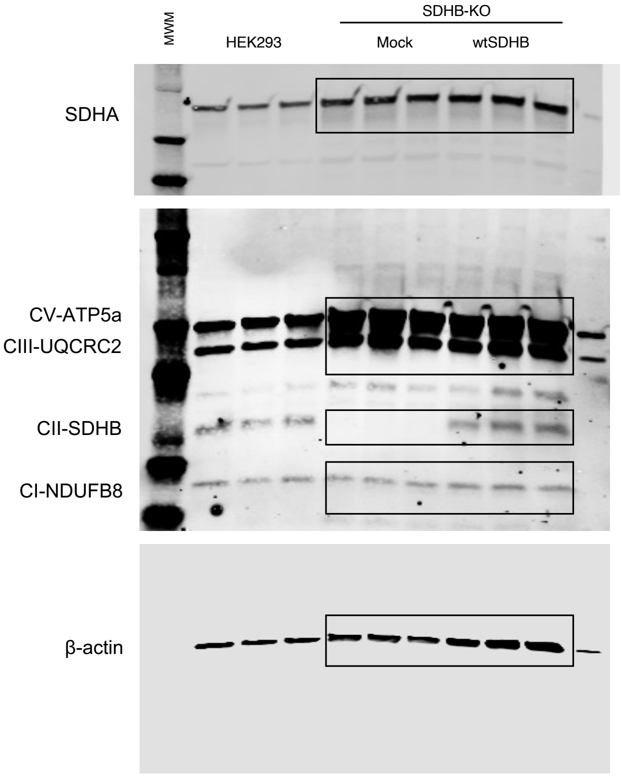

Anti-SDHA (abcam 14715), total OXPHOS Antibody (abcam110413) and β-actin (abcam179467) was blotted on the same membrane.

Supplemental Figure 3A

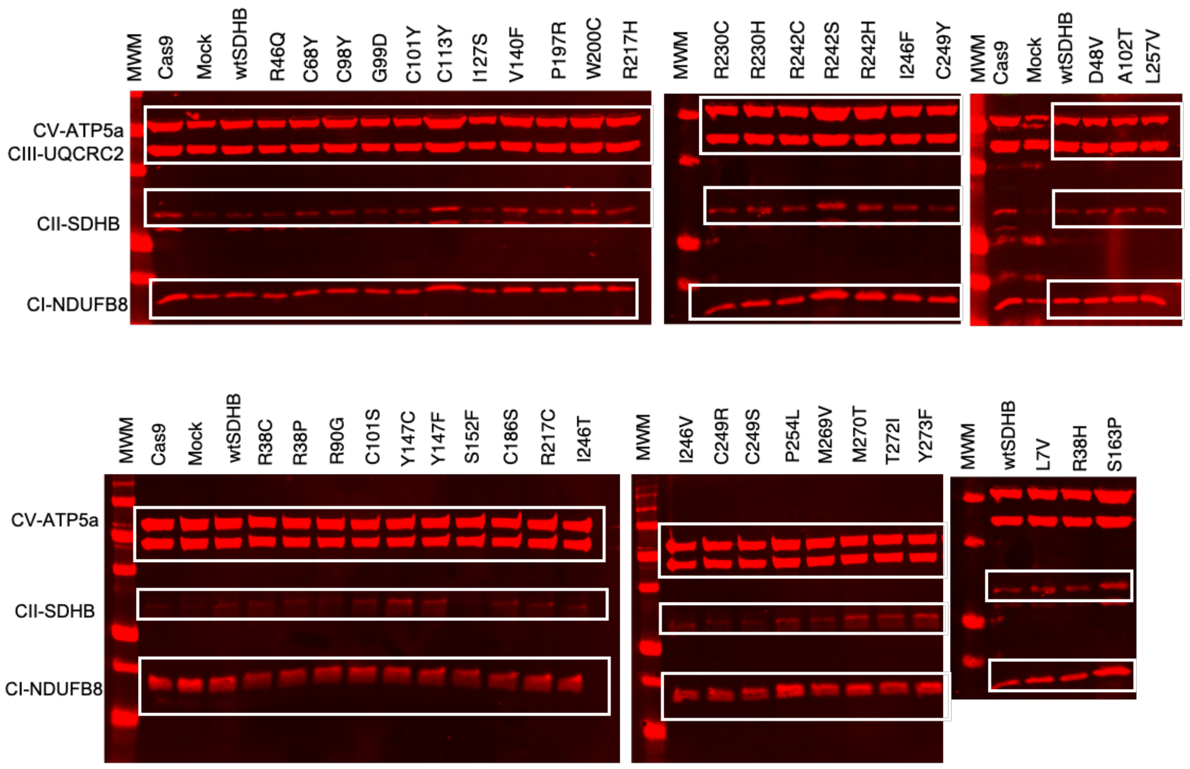

Total OXPHOS Antibody (abcam110413) was blotted on membrane. For SDHB, the blot was rescanned at the region of interest to reduce contrast from darker bands and enhance visualization of SDHB signal.

Supplemental Figure 3B

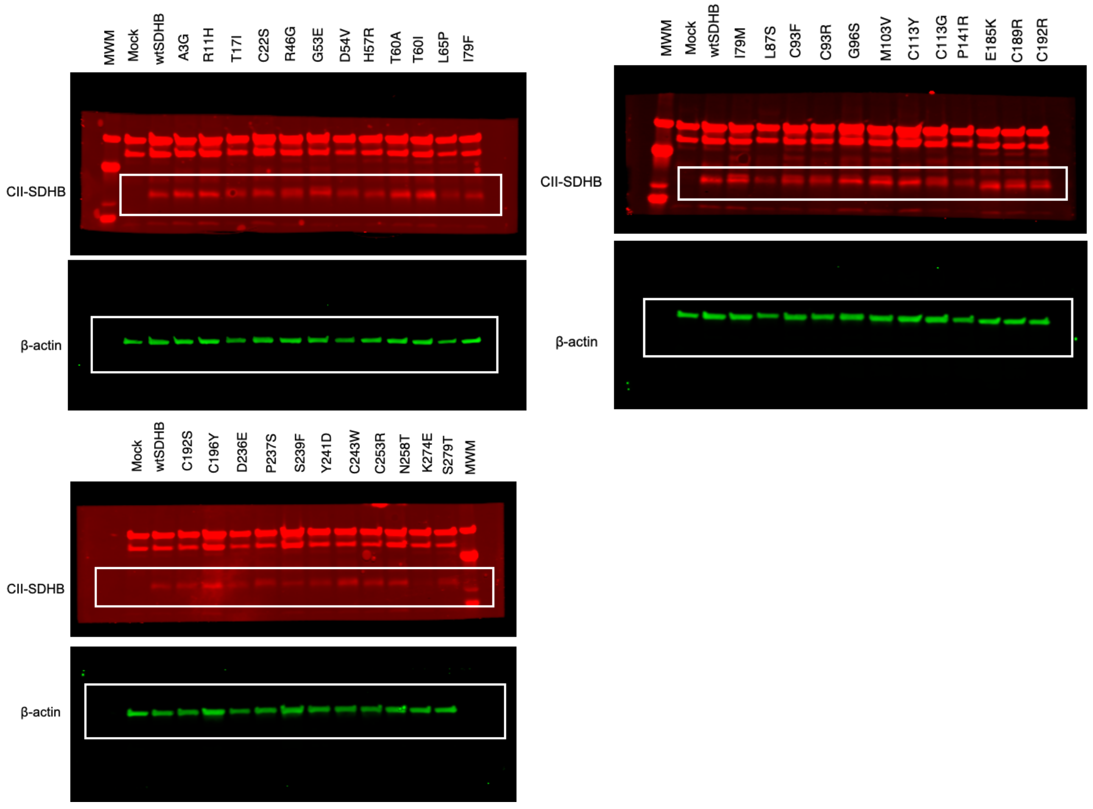

Total OXPHOS Antibody (abcam110413) and β-actin (abcam179467) was blotted on the same membrane.

Supplemental Figure 3C

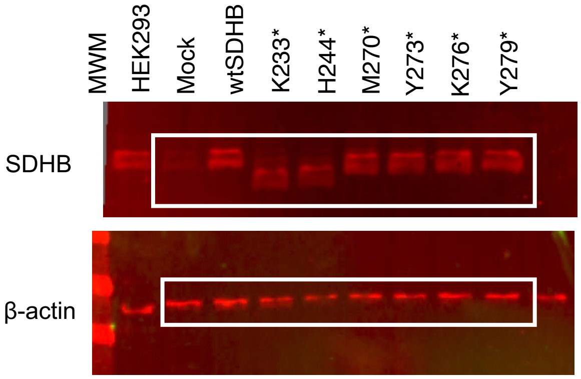

Anti-SDHB Antibody (abcam 14714) was blotted first, and then anti-β-actin (Sigma-Aldrich A5316) was re-blotted on the same membrane.
